# Poor Sleep Quality and Daytime Sleepiness in Health Professionals: Prevalence and Associated Factors

**DOI:** 10.3390/ijerph18136864

**Published:** 2021-06-26

**Authors:** Vergílio Pereira Carvalho, Kênia Alves Barcelos, Ely Paula de Oliveira, Sarah Nogueira Marins, Isabella Beatriz Silva Rocha, Daniel Ferreira Moraes de Sousa, Bruno Cabral Moreira, Gunther Abreu de Almeida, Marina Luana Silva Carneiro, Jéssica Duarte de Freitas Silva, Maria Alice Vieira de Freitas, Matias Noll, Carolina Rodrigues Mendonça

**Affiliations:** 1 UniRV-GO, Faculdade de Medicina, Universidade de Rio Verde, Rio Verde, Goiás 75901-970, Brazil; keniabarcelos@unirv.edu.br (K.A.B.); dr.ely@hotmail.com (E.P.d.O.); sarahmarins9@gmail.com (S.N.M.); isabella_bsr@hotmail.com (I.B.S.R.); danielfmds@hotmail.com (D.F.M.d.S.); bcmbruno00@gmail.com (B.C.M.); gunther.almeida@gmail.com (G.A.d.A.); marinalscarneiro@gmail.com (M.L.S.C.); jessicaduartefsrv@gmail.com (J.D.d.F.S.); licinhavf@gmail.com (M.A.V.d.F.); 2 Postgraduate Program in Health Sciences, Faculdade de Medicina, Universidade Federal de Goiás, Goiás 74605-050, Brazil; matias.noll@ifgoiano.edu.br (M.N.); carol_mendonca85@hotmail.com (C.R.M.); 3 Instituto Federal Goiano (IF Goiano), Goiás 74270-040, Brazil

**Keywords:** sleep, Pittsburgh Sleep Quality Index (PSQI), Epworth Sleepiness Scale (ESS), healthcare professionals

## Abstract

This study aimed to examine the prevalence rates of poor quality sleep and daytime sleepiness in health professionals and their association with socioeconomic, lifestyle, and anthropometric factors and psychosocial work conditions. This cross-sectional study was performed with health professionals from various public and private hospitals in Rio Verde, Goiás, Brazil. Daytime sleepiness and sleep quality were assessed using the Epworth Sleepiness Scale and the Pittsburgh Sleep Quality Index, respectively. The variables were analyzed using multiple hierarchical Poisson regression in the statistical package Stata version 13.0. A total of 244 health professionals participated in this study (women, 78.28%). The rates of abnormal daytime sleepiness and poor sleep quality were 50.41% (*n* = 123) and 55.74% (*n* = 136), respectively. Reduced physical activity was associated with poor sleep quality (prevalence ratio (PR) = 1.32; 95% confidence interval (CI) 1.02–1.70, *p* = 0.035). Age between 20 and 29 years (PR = 2.59; 95% CI 1.37–4.91, *p* = 0.021) and 30 and 49 years (PR = 2.09; 95% CI 1.12–3.91, *p* = 0.021), as well as excessive alcohol consumption (PR = 1.29; 95% CI 1.01–1.66, *p* = 0.048), were risk factors for daytime sleepiness. Conversely, adequate bodyweight (PR = 0.52; 95% CI 0.33–0.82, *p* = 0.005) was considered a protective factor. The present findings suggest high rates of abnormal daytime sleepiness and poor sleep quality among healthcare professionals. We emphasize the importance of physical activity, adequate weight, and healthy habits for better quality sleep and reduced daytime sleepiness.

## 1. Introduction

Sleep is a fundamental biological process that determines human health and performance [1]. Adequate sleep levels are responsible for restoring energy and promoting healing, as sleep interacts with the immune system. Further, sleep regulation impacts brain function and behavior [1]. Consequently, sleep loss directly affects the human body’s physiology [2]. The short-term repercussions of interrupted sleep include increased stress levels; somatic problems; reduced quality of life; emotional suffering; mood disorders and other mental health problems; deficits in cognition, memory, and performance; and behavioral problems in healthy individuals [2]. 

Health professionals are responsible for guiding their patients’ health choices; however, many of these professionals may not practice self-care or maintain healthy lifestyles. [3]. Long working hours, reduced sleep opportunities with minimal recovery time, shift work, and work stress contribute to impaired physical, cognitive, and emotional functioning [4,5]. The harmful effects of poor sleep quality and drowsiness in healthcare professionals include reduced cognitive skills [6], reduced psychomotor performance and productivity [7], and increased risk of causing medical errors and compromising patient health and safety [8].

Previous studies have examined the quality of sleep and levels of daytime drowsiness in health professionals [9,10,11,12,13,14,15], particularly in the context of the ongoing coronavirus disease 2019 pandemic [9,10,11,12,13,14]. However, data on factors affecting nighttime sleep and daytime drowsiness in health professionals across professional categories remain limited [15,16]. Given the relative paucity of previous research on associated factors in health professionals, this study aimed to examine the rates of poor quality sleep and daytime sleepiness among health professionals and the impacts of socioeconomic, lifestyle, and anthropometric factors along with psychosocial work conditions were considered. This information is vital for the adoption of appropriate management strategies in the hospital environment. We hypothesized that socioeconomic, lifestyle, and anthropometric factors, along with psychosocial work conditions, influence sleep quality and daytime sleepiness among health professionals.

## 2. Materials and Methods

### 2.1. Study Design and Participants

This cross-sectional study was conducted between May 2018 and February 2019 with 244 health professionals from various public and private hospitals in the city of Rio Verde, Goiás, Brazil, including Rio Verde University Hospital (HMU-RV), Dr. Gordon Presbyterian Hospital, Santa Terezinha Hospital, and Rio Verde Emergency Care Unit (UPA-RV), Goiás. 

### 2.2. Approval of the Bioethics Commission 

This study was approved by the Research Ethics Committee of the University of Rio Verde under protocol numbers 2.677.090/2018 and CAAE 88088418.0.0000.5077. All participants signed an informed consent form.

### 2.3. Criteria and Inclusion and Exclusion

Male and female health professionals (doctors, biomedical scientists, nurses, nursing technicians, laboratory technicians, pharmacists, speech therapists, nutritionists, physiotherapists, psychologists, social workers, and radiology technicians) aged 20–70 years were included in this study. Professionals who experienced an acute or chronic illness that could interfere with sleep quality in the preceding month and professionals who declined consent were excluded from this study.

### 2.4. Data Collection Procedures

Data were collected through a standardized and piloted questionnaire that was administered through interviews.

Independent variables included socioeconomic, lifestyle, and anthropometric factors, as well as psychosocial work conditions. Socioeconomic variables included sex, age, ethnicity, marital status, profession, education, and family income relative to minimum wage, as defined in Brazil’s 2019 Annual Budget Law. Lifestyle variables included smoking (yes/no), alcohol consumption at least three times a month (yes/no), and physical activity at least three times a week (yes/no). Psychosocial work conditions referred to the number of weekly working hours. Bodyweight and height information were used to calculate body mass index (BMI = body mass (kg)/height (m)^2^). Following the recommendations of the World Health Organization (WHO, 2000) for BMI, individuals were classified as low weight (BMI < 18.5 kg/m^2^), adequate weight (BMI ≥ 18.5 and <25.0 kg/m^2^), overweight (BMI ≥ 25.0 and <30.0 kg/m^2^), or obese (BMI ≥ 30.0 kg/m^2^).

The outcome variables were daytime sleepiness and sleep quality. Sleepiness was assessed using the Epworth Sleepiness Scale (ESS). A score of 1–6 points represented normal sleepiness, 7–8 points indicated average sleepiness, and 9–24 points referred to abnormal sleepiness [17]. Sleep quality was assessed using the Pittsburgh Sleep Quality Index (PSQI), validated for the Portuguese language [18]. The PSQI is a self-administered questionnaire that assesses sleep quality and disturbances in the preceding month. In the questionnaire, 19 individual items translate into seven component scores: subjective sleep quality, sleep latency, sleep duration, habitual sleep efficiency, sleep disorders, use of sleeping medication, and daytime dysfunction. All component scores were weighted equally on a scale ranging from 0 (no difficulty) to 3 (severe difficulty). The component scores were added to produce an overall PSQI score, ranging from 0–21 points, with a higher score indicating poorer sleep quality. An overall PSQI score of ≥5 points was indicative of poor sleep quality among young adults [19]. Thus, two outcomes were considered: abnormal sleepiness (yes/no) and poor sleep quality (yes/no).

### 2.5. Statistical Analysis

The statistical package Stata version 16.0 (Stata Corp LP, College Station, TX, USA) was used for data analyses. Statistical significance was established at the cutoff value of *p* < 0.05. Descriptive analyses are presented as absolute counts (n) and relative frequencies (%), together with the mean and standard deviation. The Chi-square test (χ2) or Fisher’s exact test were used in the bivariate analysis. Poisson regression was used to calculate the prevalence ratio (PR) and 95% confidence interval (CI), while *p*-values were obtained using the Wald test [20]. Variables with *p*-values of <0.20 in the bivariate analysis were included in the multiple Poisson regression analyses, with robust variance based on a hierarchical model [21]. The independent variables in this hierarchical analysis were grouped into four categories: (I) socioeconomic variables (age and ethnicity), (II) lifestyle (alcohol consumption and physical activity), (III) psychosocial work condition (working hours/week), and (IV) anthropometric findings (BMI (kg/m^2^)) [22,23]. 

## 3. Results

A total of 244 health professionals participated in this study (women, 78.28%), with an average age of 37.1 ± 0.6 years. Most of the sample consisted of nursing technicians (*n* = 135; 55.33%), nurses (*n* = 28; 11.48%), doctors (*n* = 20; 8.20%), and radiology technicians (*n* = 17; 6.97%). Pharmacy assistants and physiotherapists represented 3.69% (*n* = 9) of the sample for each professional category; biomedical and social workers represented 2.87% (*n* = 7); and a laboratory technician, a nutritionist and a psychologist represented 1.23% (*n* = 3) of the sample for each professional category.

**Figure 1:**
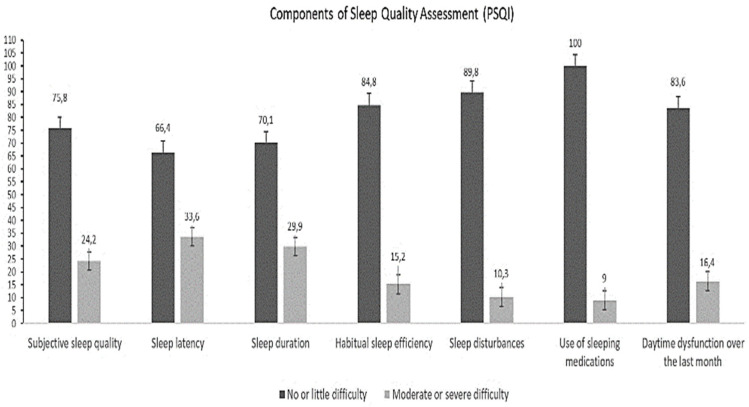
Components of the Pittsburgh Sleep Quality Index in healthcare professionals.

The average daytime sleepiness in health professionals according to the ESS was 8.7 ± 0.3. Eighty-five (34.84%) health professionals reported normal sleep, 36 (14.75%) reported average daytime sleepiness, and 123 (50.41%) reported abnormal daytime sleepiness. Regarding sleep quality, 108 (44.26%) health professionals experienced good sleep quality and 136 (55.74%) had poor sleep quality. The average sleep duration was 5.66 ± 0.19 h per day, which indicated poor sleep quality. The components of the PSQI for health professionals are shown in Figure 1.

Data on the rates of sleepiness and poor sleep quality and their association with socioeconomic, lifestyle, and anthropometric factors, as well as psychosocial working conditions, in health professionals are presented in Table 1. Bivariate analysis indicated that abnormal sleepiness was associated with individuals aged 30–49 years and those with excessive alcohol consumption (Table 1). 

Variables with *p*-values < 0.20 were included in the multivariate analysis (Table 2). Thus, after adjusting the hierarchical multivariate model, poor sleep quality was associated with poor physical activity (PR = 1.32; 95% CI 1.02–1.70); meanwhile, abnormal drowsiness was associated with age (20–29 years (PR = 2.59; 95% CI 1.37–4.91) and 30–49 years (PR = 2.09; 95% CI 1.12–3.91)), excessive alcohol consumption (PR = 1.29; 95% CI 1.00–1.66), adequate weight (PR = 0.52; 95% CI 0.33–0.82), and being overweight (PR = 0.55; 95% CI 0.34–0.89).

## 4. Discussion

The main results of this study indicated a high prevalence of abnormal daytime sleepiness and poor sleep quality among health professionals. In addition, abstaining from physical activity was considered a risk factor for poor sleep quality among health professionals. Age and excessive alcohol consumption were considered risk factors for abnormal sleepiness, and adequate weight was considered a protective factor. These results are important for encouraging good daily living habits, which can improve the quality of sleep and reduce daytime sleepiness in health professionals.

These findings are consistent with those of previous studies [9,12,13,14], in which poor sleep quality in shift workers, in particular, was responsible for a deterioration in their quality of life [16,24]. In addition, it is important to highlight that chronic insufficient sleep, whether due to unrecognized sleep disorders, or prolonged hours of work and circadian misalignment, is a critical risk factor for burnout [25]. Although this study was performed in 2018 and 2019, before the coronavirus disease 2019 pandemic, this topic has been the subject of much research in the present context [10,11]. In Brazil, the prevalence of impaired sleep quality among physicians reached 70% as of 2020 [26]. This is because the pandemic has had a major impact on the work environment, particularly for frontline health professionals at increased risk of infection, with an overwhelming workload and an isolating environment. These factors may increase the symptoms of anxiety and depression, which are associated with sleep disorders, including insomnia [27].

Our findings indicate that abstaining from physical activity is associated with poor sleep quality. There is an indisputable consensus that sufficient sleep and adequate exercise are essential for maintaining good health [28]. Conversely, inadequate amounts of exercise and insufficient sleep may be harmful [2,28]. The present findings emphasize the need to encourage physical activity among health workers to help prevent and treat sleep disorders [29]. 

In this study, age and excessive alcohol consumption were associated with daytime sleepiness. Sleep problems due to age are difficult to solve because they cannot be controlled [16]; however, lifestyle habits, such as alcohol consumption, may be regulated. One hypothesis that may help explain the present findings regarding sleepiness in young adults is that they may have fewer hours of sleep than older adults, either due to the increased workload or the number of hours dedicated to evening entertainment. Therefore, the adoption of healthy lifestyle habits may significantly impact the quality of life and work of young health professionals [30]. 

This study also revealed that adequate weight might protect against drowsiness, further emphasizing the need to adopt healthy lifestyles among health workers. Controlling the bodyweight of day workers may improve their sleep quality. Weight loss effectively prevents various obesity-related diseases and may help resolve sleep disorders in obese workers [16]. Surprisingly, in our study, being overweight emerged as protective against drowsiness. This finding was unexpected; we could not find other studies reporting this association or speculating on the underlying mechanism. The prevalence of overweight and obesity in our sample was 33.61 and 18.85%, respectively. The relationship between daytime sleepiness and obesity has been described in the literature and is mainly associated with metabolic and psychological factors [31,32,33]. 

### Limitations and Strengths

Our study has some limitations. First, it was a cross-sectional study, precluding any meaningful discussions on causality. Second, this study was performed with participants sampled from a municipality in the interior of Goiás; therefore, the extrapolation of the results to the rest of the Brazilian population requires caution. Third, although our sample comprised different professional categories, most of the participants were nursing technicians; therefore, the study results primarily reflect those of nursing technicians. Fourth, a large part of our sample consisted of professionals aged 30–49 years, the largest age group of young adults employed in Brazil. Fifth, we considered the results of hours worked in a single hospital and a workload cutoff point of 30 h per week. However, it is important to consider that many professionals work on shifts and in more than one job. Sixth, we did not investigate professionals’ commuting time. Many people spend much time on public transport, which can decrease the time available for sleep. However, this study had several strengths. First, it involved a relatively large sample of health professionals. Second, we used scales that are internationally recognized and validated for Brazil. Third, this study was the first to investigate the different factors associated with sleep quality and daytime sleepiness among different health professional groups. Therefore, the results of this study are essential for establishing specific interventions that can improve the quality of sleep and reduce daytime sleepiness in health professionals. This study provides a basis for further studies on the associated factors. We recommend that future studies explore the following variables in-depth: physical activity practices, alcohol consumption, smoking, and time using public transport for commuting between home and work.

## 5. Conclusions

This study found high prevalence rates of abnormal daytime sleepiness and poor sleep quality among healthcare professionals. We emphasize the importance of physical activity, proper weight maintenance, and adopting healthy habits that may contribute to a better quality of sleep and reduce the likelihood of daytime sleepiness.

## Figures and Tables

**Table 1:** Prevalence and bivariate analysis of the association between poor sleep quality and daytime sleepiness and socioeconomic, lifestyle, and anthropometric factors, as well as psychosocial work conditions, in health professionals (*n* = 244).

		Poor Sleep Quality			Abnormal Drowsiness		
Variable	Frequency *n* (%)	Prevalence *n* (%)	PR (95% CI)	*p*	Prevalence *n* (%)	PR (95% CI)	*p*
Sex				0.639			0.826
Male	53 (21.72)	28 (52.8)	1		26 (21.14)	1	
Female	191 (78.28)	108 (56.54)	1.07 (0.81–1.42)		97 (78.86)	1.04 (0.76–1.41)	
Age (years)				0.0677			**0.0081**
20–29	61 (25.00)	29 (21.32)	0.67 (0.47–0.95)		39 (31.71)	2.56 (1.31–4.99)	
30–49	155 (63.52)	87 (63.97)	0.79 (0.60–1.03)		77 (62.60)	1.99 (1.02–3.85)	
50 or more	28 (11.48)	20 (14.71)	1		7 (5.69)	1	
Skin color				0.1289			0.6151
White	69 (28.28)	31 (22.79)	0.72 (0.50–1.01)		32 (26.02)	0.83 (0.57–1.20)	
Mixed-race	132 (54.10)	78 (57.35)	0.94 (0.72–1.23)		67 (54.47)	0.91 (0.66–1.25)	
Black	43 (17.62)	27 (19.85)	1		24 (19.51)	1	
Lives with a partner				0.2882			0.6458
No	115 (47.33)	68 (50.37)	1		60 (48.78)	1	
Yes	128 (52.67)	67 (49.63)	0.88 (0.71–1.11)		63 (51.22)	0.94 (0.74–1.21)	
Profession				0.8952			0.7590
Technical level	157 (64.34)	88 (64.71)	1.02 (0.80–1.29)		78 (63.41)	0.96 (0.74–1.24)	
Higher level	87 (35.66)	48 (35.29)	1		45 (36.59)	1	
Family income ^1^				0.5748			0.2036
1–3 salaries	52 (21.31)	33 (24.26)	1.17 (0.83–1.63)		23 (18.70)	0.70 (0.48–1.02)	
3–6 salaries	96 (39.34)	50 (36.76)	0.96 (0.69–1.33)		47 (38.21)	0.77 (0.57–1.05)	
6–9 salaries	50 (20.49)	28 (20.59)	1.03 (0.71–1.48)		24 (19.51)	0.76 (0.53–1.09)	
More than 10	46 (18.85)	25 (18.38)	1		29 (23.58)	1	
Smoker				0.2069			0.3416
No	228 (93.44)	125 (91.91)	1		117 (95.12)	1	
Yes	16 (6.56)	11 (8.09)	1.25 (0.88–1.78)		6 (4.88)	0.73 (0.38–1.39)	
Alcohol consumption				0.9094			**0.0236**
No	123 (50.41)	67 (49.26)	1		52 (42.28)	1	
Yes	123 (50.41)	69 (50.74)	1.01 (0.81–1.27)		71 (57.72)	1.34 (1.04–1.73)	
Physical activity				0.0133			0.1337
No	149 (61.07)	93 (68.38)	1.38 (1.06–1.78)		81 (65.85)	1.23 (0.94–1.61)	
Yes	95 (38.93)	43 (31.62)	1		42 (34.15)	1	
Workload (hours/week)				0.1741			0.3278
≤30	27 (11.07)	18 (13.24)	1		11 (8.94)	1	
>30	217 (88.93)	118 (86.76)	0.82 (0.61–1.09)		112 (91.06)	1.27 (0.79–2.03)	
Body mass index (kg/ m^2^)				0.1729 *			0.1131 *
Low weight	5 (2.05)	4 (2.94)	1		4 (3.25)	1	
Adequate weight	111 (45.49)	55 (40.44)	0.62 (0.38–1.00)		53 (43.09)	0.59 (0.37–0.96)	
Overweight	82 (33.61)	49 (36.03)	0.75 (0.47–1.20)		39 (31.71)	0.59 (0.36–0.97)	
Obese	46 (18.85)	28 (20.59)	0.76 (0.46–1.25)		27 (21.95)	0.73 (0.44–1.2)	

CI: confidence interval; PR: adjusted prevalence ratio. *p*-values of <0.05 were considered statistically significant (indicated by bold text). The Wald test was used to calculate all “*p*”-values, except when frequencies were below five, in which case Fisher’s exact test (*) was used. Variables with *p*-values < 0.20 were subsequently included in multiple hierarchical Poisson regression. ^1^ The minimum wage was BRL954 as defined in the 2019 Annual Budget Law Project. This amounts to USD182.54. An overall PSQI score of ≥5 points was indicative of poor sleep quality.

**Table 2:** Multiple analysis of the association of poor sleep quality and drowsiness and risk factors in health professionals (*n* = 244).

	Poor Sleep Quality		Abnormal Drowsiness	
Variable	PR (95% CI)	*p*	PR (95% CI)	*p*
**First Level**				
Age (years)		0.155		**0.021**
20–29	0.73 (0.51–1.04)		2.59 (1.37–4.91)	
30–49	0.82 (0.63–1.08)		2.09 (1.12–3.91)	
50 or more	1		1	
Skin color		0.838		-
White	0.77 (0.54–1.10)		-	
Mixed race	0.97 (0.73–1.29)		-	
Black	1		-	
**Second Level**				
Alcohol consumption		-		**0.048**
No	-		1	
Yes	-		1.29 (1.00–1.66)	
Physical activity		**0.035**		0.137
No	1.32 (1.02–1.70)		1.21 (0.94–1.56)	
Yes	1		1	
**Third Level**				
Amount of work (hours/week)		0.27		-
≤30	1		-	
>30	0.85 (0.63–1.14)		-	
**Fourth Level**				
Body mass index (kg/m^2^)		0.240		**0.005**
Low weight	1		1	
Adequate weight	0.65 (0.39–1.07)		0.52 (0.33–0.82)	
Overweight	0.77 (0.47–1.26)		0.55 (0.34–0.89)	
Obese	0.74 (0.45–1.22)		0.68 (0.41–1.12)	

CI: confidence interval; PR: prevalence ratio. The Wald test was used to calculate all *p*-values. *p*-values of <0.05 were considered statistically significant (indicated by bold text).

## Data Availability

The data presented in this study are available on request to the authors. The data are not publicly available due to privacy reasons.
